# Mice with an induced mutation in collagen 8A2 develop larger eyes and are resistant to retinal ganglion cell damage in an experimental glaucoma model

**Published:** 2012-05-01

**Authors:** Matthew R. Steinhart, Frances E. Cone, Cathy Nguyen, Thao D. Nguyen, Mary E. Pease, Oliver Puk, Jochen Graw, Ericka N. Oglesby, Harry A. Quigley

**Affiliations:** 1 Glaucoma Center of Excellence, Wilmer Ophthalmological Institute, Johns Hopkins University, Baltimore, MD; 2 Department of Mechanical Engineering, Johns Hopkins University, Baltimore, MD; 3 Institute of Developmental Genetics, Helmholtz Center, Munich, Germany

## Abstract

**Purpose:**

To study susceptibility to glaucoma injury as it may be affected by mutations in ocular connective tissue components.

**Methods:**

Mice homozygous for an N-ethyl-N-nitrosourea induced G257D exchange (Gly to Asp) missense mutation (Aca23) in their collagen 8A2 gene were studied to measure intraocular pressure (IOP), axial length and width, number of retinal ganglion cells (RGC), and inflation responses. Three month old homozygous Aca23 mutant and wild type (WT) mice had 6 weeks exposure to elevated IOP induced by polystyrene microbead injection. Additional Aca23 and matched controls were studied at ages of 10 and 18 months.

**Results:**

Aca23 mice had no significant difference from WT in IOP level, and in both strains IOP rose with age. In multivariable models, axial length and width were significantly larger in Aca23 than WT, became larger with age, and were larger after exposure to glaucoma (n=227 mice). From inflation test data, the estimates of scleral stress resultants in Aca23 mice were similar to age-matched and younger WT C57BL/6 (B6) mice, while the strain estimates for Aca23 were significantly less than those for either WT group in the mid-sclera and in some of the more anterior scleral measures (p<0.001; n=29, 22, 20 eyes in Aca23, older WT, younger WT, respectively). With chronic IOP elevation, Aca23 eyes increased 9% in length and 7% in width, compared to untreated fellow eyes (p<0.05, <0.01). With similar elevated IOP exposure, WT eyes enlarged proportionately twice as much as Aca23, increasing in length by 18% and in nasal—temporal width by 13% (both p<0.001, Mann–Whitney test). In 4 month old control optic nerves, mean RGC axon number was not different in Aca23 and WT (46,905±7,592, 43,628±11,162, respectively; p=0.43, Mann–Whitney test, n=37 and 29). With chronic glaucoma, Aca23 mice had a mean axon loss of only 0.57±17%, while WT mice lost 21±31% (median loss: 1% versus 10%, n=37, 29, respectively; p=0.001; multivariable model adjusting for positive integral IOP exposure).

**Conclusions:**

The Aca23 mutation in collagen 8α2 is the first gene defect found to alter susceptibility to experimental glaucoma, reducing RGC loss possibly due to differences in mechanical behavior of the sclera. Detailed study of the specific changes in scleral connective tissue composition and responses to chronic IOP elevation in this strain could produce new therapeutic targets for RGC neuroprotection.

## Introduction

Glaucoma is the second leading cause of blindness worldwide [1], and the risk factors for open angle glaucoma (OAG), its most prevalent form, include structural features of the eye, such as greater axial length, thinner central cornea, and larger optic disc diameter [2]. Some of these risk factors are clearly related to the connective tissue structures in the eye. There are 14 chromosomal loci and mutations of three genes that have been identified among primary OAG patients: namely, myocilin (*MYOC*), optineurin (*OPTN*), and WD repeat domain 36 (*WDR36*) [3]. The pathogenetic pathway by which these mutations act has not been specified in detail. Both higher mean IOP [4] and greater IOP fluctuation [5] increase OAG injury, related to IOP-generated stress at the optic nerve head (ONH). IOP-related stress in the cornea and sclera is transmitted to the ONH at its periphery [6], which is thought to be directly involved in retinal ganglion cell (RGC) death by apoptosis [7]. The linkage between activation of detrimental processes by IOP-induced stress and RGC death represents an important area for development of new treatments.

To utilize the power of mouse genetics in the elucidation of glaucoma pathogenesis, numerous models have been developed with either experimental IOP elevation [8-17], spontaneous IOP elevation [18-20], or spontaneous RGC death [21,22]. Mammalian eyes, including those of mice, that are subjected to experimental increases in IOP have neuronal, glial, and associated tissue alterations that are phenotypically similar to human glaucoma [23-25]. Furthermore, lowering of IOP slows the progressive loss of RGCs in both animal and human glaucoma [26,27]. While mouse eyes differ in details of ONH anatomy from primates, they share the site of glaucoma injury and the selective death of RGC. Jakobs et al. [28] demonstrated that astrocytes in the mouse ONH simulate the structure of the collagenous lamina cribrosa in primate/human eyes, potentially transferring scleral wall tension to ONH axons and capillaries. The mouse sclera has collagens, elastin, and a general molecular content that is similar to human sclera [29], though its thickness and diameter are 10 times smaller than those of human eyes [30].

Collagen 8 is a short chain collagen found in the extracellular matrix of several tissues, including Descemet’s membrane in the cornea [31-34], the sclera, and the optic nerve [35-37]. Defects in collagen 8α2 are associated with an early onset form of Fuchs corneal dystrophy [38,39]. Duplication of the corresponding mutation for Fuchs dystrophy in the mouse gene replicates abnormalities in corneal endothelium and Descemet’s membrane seen in affected humans [40]. Targeted knockout of both collagen 8α1 and 8α2 in mice produces a thinner than normal cornea and an increased anterior chamber depth [41]. An induced mutation in collagen 8α2 leading to a G257D amino acid exchange affecting a highly conserved glycine residue in the collagenous domain produced thinner cornea and enlarged anterior chambers in mice homozygous for the defect (the Aca23 mouse) [42]. Visual acuity was normal and the histology of the retina and optic nerve were normal.

We hypothesized that mouse eyes with scleral abnormalities leading to increased axial length would be differentially susceptible to glaucoma damage. The Aca23 mouse has both longer and wider than normal eyes and served as a test of this hypothesis. There is some evidence that both thinner cornea and defects in collagen 8 may be associated with human OAG. The Ocular Hypertension Treatment Study pointed to an association between thinner central cornea thickness (CCT) and the incidence of OAG [43]. An association was found between missense mutations in the collagen 8α2 gene and thinner CCT in human glaucoma patients [44]. Genome wide association study of residents of Singapore also found potential linkage between thinner CCT and a variant in the collagen 8α2 gene [45]. We therefore studied the Aca23 mouse eye in detail, with aging, and after induction of chronic IOP elevation with polystyrene microbead injection into the anterior chamber.

## Methods

### Animals

All animals were treated in accordance with the ARVO Statement for the Use of Animals in Ophthalmic and Vision Research, using protocols approved and monitored by the Johns Hopkins University School of Medicine Animal Care and Use Committee. B6 wild type (WT) mice were obtained from Jackson Laboratories, Bar Harbor, ME. The Aca23 mutant mice were rederived from heterozygotic Aca23 mice from the laboratory of Drs. Puk and Graw [42]. Aca23 semen fertilized WT B6 oocytes in vitro. Progeny that were heterozygous for the Aca23 mutation were crossed to produce mice homozygous for the mutation and age-matched WT litter mate controls. Mice were genotyped with primers directed at the mutation site using PTC-225 thermocycler (MJ Research, Waltham, MA). Polymerase chain reaction products were digested using the restriction enzyme Hpy188III and analyzed by electrophoresis on a 2% agarose gel to confirm the presence of the G257D exchange (Gly to Asp) missense mutation (Aca23) in the collagen 8A2 gene. We studied 425 control or fellow eyes and 67 glaucoma eyes from WT B6 mice and 209 control or fellow eyes and 37 glaucoma eyes from Aca23 mutants.

### Bead injection glaucoma

For anterior chamber injections to produce glaucoma, mice were anesthetized by intraperitoneal injection of general anesthesia, 50 mg/kg of ketamine, 10 mg/kg of xylazine, and 2 mg/kg of acepromazine. Additional topical anesthesia was provided, when using general anesthesia, by 0.5% proparacaine hydrochloride eye drops (Akorn Inc., Buffalo Grove, IL). In the injection protocol (the 4 + 1 method) [11], we injected 2 µl of 6 µm diameter beads, then 2 µl of 1 µm diameter beads (Polybead Microspheres®; Polysciences, Inc., Warrington, PA), followed by 1 µl of viscoelastic compound (10 mg/ml sodium hyaluronate, Healon; Advanced Medical Optics Inc., Santa Ana, CA) through a glass cannula pulled to a tip diameter of 50 µm connected by polyethylene tubing to a Hamilton syringe (Hamilton, Inc., Reno, NV). We estimate the final concentration as 3×10^6^ beads per µl for 6 µm beads, and 1.5×10^7^ beads per µl for 1 µm beads.

IOP measurements for bead injected animals were completed before injection, at 10 min after injection, 3 days, 1 week, and weekly to sacrifice at 6 weeks after injection. For IOP measurements, mice were anesthetized by inhalation of isoflurane using the RC^2^- Rodent Circuit Controller (VetEquip, Inc., Pleasanton, CA) [11]. The instrument supplied oxygen from an attached tank mixed with isoflurane, delivering 2.5% of isoflurane to the animal. IOP measurements were made using the TonoLab tonometer (TioLat, Inc., Helsinki, Finland), recording the mean of 6 readings with optimal variability grade.

### Sacrifice, axial length, and width measurements

Animals with induced glaucoma did not undergo inflation testing. They received intraperitoneal injection of general anesthesia before sacrifice by exsanguination, followed by intracardiac perfusion with 4% paraformaldehyde in 0.1 M sodium phosphate buffer (pH=7.2). Orientation of the eye was marked by a cautery mark on the superior cornea and optic nerves were removed 1.0 mm distal to the globe. The eyes were inflated to 15 mmHg with a needle connected to a fluid-filled reservoir. Length and widths were measured with a digital caliper (Instant Read out Digital Caliper; Electron Microscopy Sciences, Hatfield, PA). The length was measured from the center of the cornea to a position just temporal to the optic nerve insertion to the sclera. Nasal—temporal width and superior—inferior width were measured at the largest dimension at the equator, midway between the cornea and optic nerve.

For inflation tested animals, intraperitoneal general anesthesia and topical anesthesia were delivered and final IOP measurements performed. Eyes were enucleated and orientation was maintained by a cautery mark. After eyes were placed in 0.1 M sodium phosphate buffer (pH=7.2), the fat and muscle were removed, and the nerve was cut 1.0 mm posterior to the globe. Then, the nerve was fixed by immersion in 4% paraformaldehyde in 0.1M sodium phosphate buffer (pH=7.2). Measurements of the eye were taken before inflation testing using the digital caliper, as above. After completion of inflation testing, eyes were fixed by immersion in 4% paraformaldehyde in 0.1 M sodium phosphate buffer (pH=7.2).

### Inflation test methods and analysis

**Figure 1:**
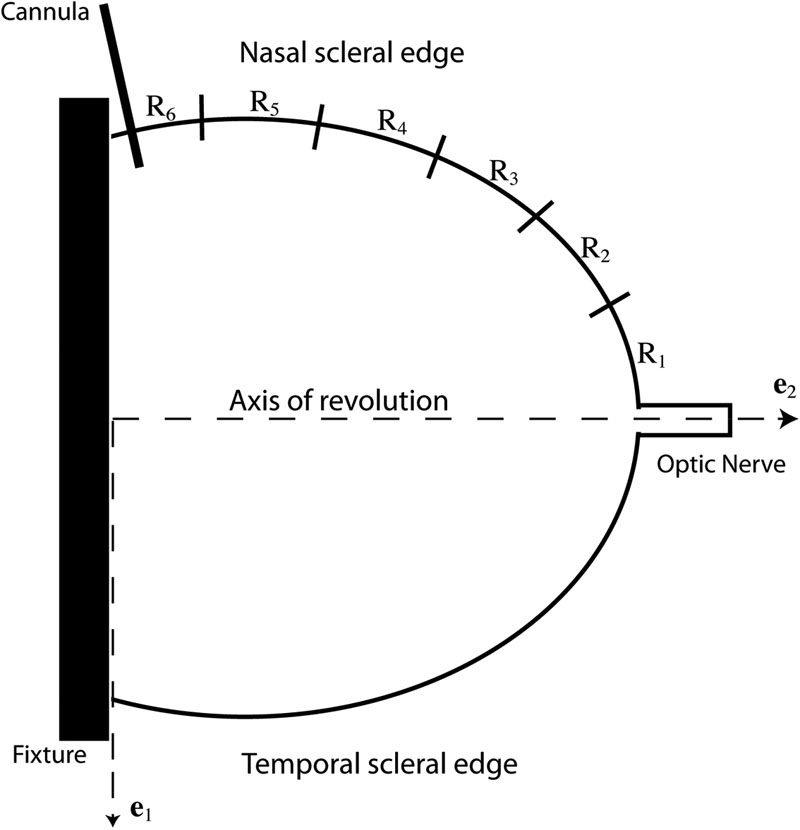
Schematic of sclera locations. This figure shows the schematic of an inflation tested left eye, where Rk indicates the labeling of regions. The positions of points along the scleral edge were calculated for the initial image (reference configuration) and for the subsequent images (deformed configurations) in the (e1, e2) Cartesian coordinate system. The axis of revolution was defined as the axis passing through the center of the optic nerve head (ONH) parallel to the axis of the ONH.

The inflation test method has been previously described in detail [46]. In brief, the eye was glued to a fixture at the limbus and inflated through pressure-controlled injection of a saline solution. Digital image correlation (DIC) was used to locate the scleral edge as seen from a superior view, extending from the fixture to the optic nerve both nasally and temporally (Figure 1). The coordinates for a series of locations along the sclera were obtained from DIC at the baseline pressure (undeformed configuration) and after displacement produced by inflation (deformed configuration). We assumed that the sclera can be described as a revolved ellipsoid, where the axis of revolution was defined as the line connecting the ONH center to the holder, the meridional direction was defined anterior-posteriorly along the scleral edge, and the circumferential direction was defined parallel to the equator. The assumption of axisymmetry was needed to calculate the strain and stress resultant response from the 2D position and displacement of the scleral edge. To calculate the stress resultants, we modeled the deformed sclera as a thin shell capable of supporting constant membrane stresses through the thickness of the scleral wall. These assumptions allowed us to estimate the stress resultants away from the ONH and fixture from the applied pressure and deformed radii of curvature using static equilibrium. The strains, on the other hand, were calculated directly from the DIC displacements.

Inflation testing used enucleated, unfixed whole mouse eyes glued with cyanoacrylate to a fixture. The anterior chamber was connected through a 30 gauge needle and tubing to a programmable transducer-pump manifold and immersed in phosphate-buffered saline at 22 °C. The preparation permitted analysis of the posterior 2/3 of the globe. A CCD video camera attached to a dissecting microscope viewed the eye from superiorly, recording sclera edge images, every 2 s, which were processed by DIC [47] software to extract the 2D displacement field of selected points along the scleral edge. The error in the displacement measurement was calculated previously as ±0.46 μm. This included contributions from the uncertainty in the pixel-distance calibration, ±0.36 μm, and the inherent error of the DIC correlation, ±0.1 μm [46]. To characterize the nonlinear, time-dependent material behavior, testing began at a reference pressure, P_0_, determined for each eye as the minimum pressure at which the sclera was no longer wrinkled, typically 6–8 mmHg. The specimen was first subjected to 2 load-unload cycles from P_0_ to 30 mmHg at a rate of 0.25 mmHg/s. The pressure was returned to P_0_ and held for 10 min after each unloading to ensure full recovery of the displacements. A ramp hold test was then conducted, at a rate of 0.25 mmHg/s from P_0_ to 30 mmHg. The specimen was held at 30 mmHg for 30 min before the pressure was brought back to P_0_ for a recovery period of 20 min. The present analysis was applied to the loading portion of the first load-unload cycle. We successfully performed inflation tests on 31 Aca23 eyes, 23 WT B6 control eyes age-matched to the Aca23 eyes (17 and 18 months old, respectively), and 21 eyes of 4 month old B6 mice. There were unsuccessful inflation tests not included here on an additional 7 Aca23 eyes, 8 WT B6 age-matched control eyes, and 13 eyes of 4 month old B6 mice. Unsuccessful inflation tests had obvious leakage from cannulation, eyes that detached from the fixture, or technical failure to complete the protocol.

**Figure 2:**
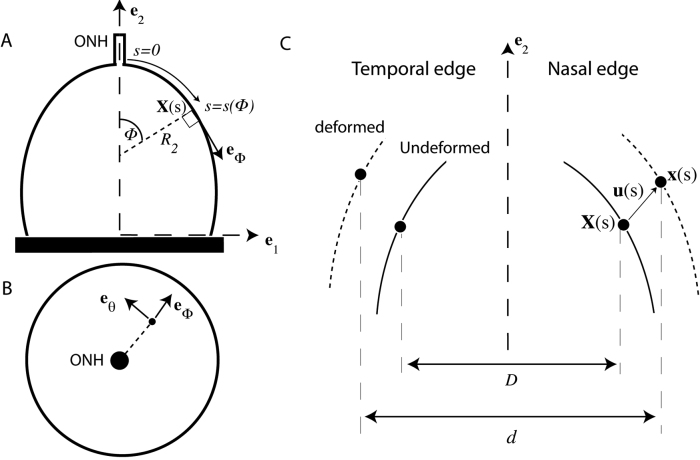
Schematic for scleral analysis. Schematics for the scleral analysis indicate the meridional and circumferential directions with angles Φ and θ, respectively, and the radius of curvature of the undeformed meridian with R2. **A**: The superior view of the sclera with curvilinear coordinates (Φ), which is used to locate a point along the scleral edge. **B**: The posterior view of the sclera indicating the two principal directions used for strains and stress calculations. **C**: The superior view of the undeformed (solid line) and deformed (dashed line) scleral edge, indicating the undeformed position, X(s), the deformed position, x(s), the displacement vector, u(s), and the diameters d and D of the deformed and undeformed cross-sections at s, respectively.

To calculate the stress resultant-strain response, 2 different coordinate systems were used - (1) a Cartesian coordinate system (**e**_1_, **e**_2_), where **e**_2_ was the axis of revolution (Figure 1 and Figure 2) a curvilinear coordinate system, *s*, following the scleral edge (Figure 2A,B). The strains in the meridional direction (Figure 2A), parameterized by the angle Φ, and in the circumferential direction, parameterized by the angle θ (Figure 2B), were measured from the DIC displacement field using the assumptions of a thin shell of revolution. To obtain an analytical description of the scleral edge, ellipses were fitted to the coordinates of the reference image taken at the baseline pressure and to the coordinates of the deformed images taken at up to 7 subsequent pressure steps. The coordinates of the deformed positions **x**(*s*) were given by:

x(s)=X(s)+u(s),             1

where **X**(*s*) is the coordinates of the points in the Cartesian coordinate system in the reference image, and **u**(*s*) is the corresponding displacement vector (Figure 2C). The experiments measured the Cartesian components of the displacement *u_1_* and *u_2_*. To determine the strains, the displacement components were fitted to a fourth order polynomial as a function of a parameter representing the counterclockwise angle from the major axis of the fitted ellipse (submitted), using the Matlab function polyfit. The analytical displacements were used to calculate the Green-Lagrange strain component in the meridional direction as follows:

$$\begin{array}{l} E_{\Phi }(s)=\frac{1}{2}\left(\frac{dx(s)}{ds}.\frac{dx(s)}{ds}-1\right)\\ =\frac{dX}{ds}.\frac{du}{ds}+\frac{1}{2}\frac{du}{ds}.\frac{du}{ds} \end{array}$$, 2

The meridional strain, *E_Φ_*, describes the elongation of the sclera in the anterior-posterior direction.

Circumferential strains were calculated from the ratio of the deformed diameter *d* to the undeformed diameter *D* as indicated in (Figure 2C), using the assumption of axisymmetry:

$$E_{\theta }(s)=\frac{1}{2}\left[{\left(\frac{d(s)}{D(s)}\right)}^{2}-1\right]$$, 3

To calculate the stress resultants in the circumferential and meridional directions, we assumed that away from the ONH and fixture, the shear stress and strain components were negligible compared to the normal components and that the effects of stress gradients from bending were also negligible. This allowed the sclera to be modeled as a thin shell with constant membrane stresses through the thickness [48]. The meridional and circumferential stress resultants of a thin shell of revolution subjected to an internal pressure, *p*, can be determined from equilibrium as:

$$n_{\Phi }=p\frac{r_{2}}{2},\;n_{\theta }=pr_{2}\frac{2r_{1}-r_{2}}{2r_{1}}$$, 4

where *r_2_* is the radius of curvature of the deformed meridian and *r_1_* is the transverse radius of curvature, analytically calculated from the deformed positions (submitted). The stress resultants and strains were calculated at up to 8 points located in regions 2 and 3 (Figure 1) and then averaged to provide a single stress resultant-strain curve for region 2–3; a separate stress resultant-strain curve for region 4 was calculated. We used the ratios E_θ_ /E_Φ_ and n_θ_ /n_Φ_, to describe the mechanical anisotropy of the tissue.

Among eyes with successful inflations, we were able to apply the analysis to 29 Aca23 eyes, 22 WT B6 control eyes age-matched to the Aca23 eyes (17 and 18 months of age, respectively), and 20 eyes of 4 month old B6 mice. The 4 remaining successful inflations could not be analyzed because of poor polynomial fitting of the displacement data or because of poor DIC correlations at the pressure steps of interest.

### RGC axon loss measurement

To assess RGC damage, we estimated axon loss in optic nerve cross-sections by a quantitative, sampling technique [49,50]. After initial aldehyde fixation, the optic nerve was removed and post-fixed in 1% osmium tetroxide, dehydrated in alcohol and stained with 1% uranyl acetate in 100% ethanol for 1 h. Nerves were embedded in epoxy resin and 1 µm cross-sections were digitally imaged to measure each optic nerve area. Then, five 40×40 μm, randomly selected 100× images were made (Cool Snap camera, Metamorph Image Analysis software; Molecular Devices, Downington, PA), comprising 9% of the overall nerve area. Masked observers edited non-axonal elements from each image to estimate true axon density. The average axon density/mm^2^ was multiplied by the individual nerve area to estimate the axon number. Experimental eyes were compared to the mean axon number in pooled, fellow eye nerves to yield percent axon loss.

### RGC body loss quantification

Retinal whole mounts from Aca23 and WT were fixed with 4% paraformaldehyde, rinsed in buffer, then incubated overnight in 1:500 anti-β-tubulin (Covance, Inc., Princeton, NJ). An AlexaFluor 488 labeled secondary antibody (Invitrogen, Carlsbad, CA) was used to detect the primary antibody. Nuclei were stained with 4’,6-diamidino-2-phenylindole dihydrochloride (DAPI; Invitrogen) during the final phosphate buffered saline wash before being coverslipped with DAKO Fluorescent Mounting Medium (DAKO, Carpenteria,CA). Samples were imaged with a Zeiss LSM 510 Meta Confocal Microscope (Zeiss MicroImaging, Thornwood, NY). Four 40× images were taken in the superior and in the temporal retina. Manual quantification of RGC identified as positive for β-tubulin, as well as all RGC layer cells labeled with DAPI was performed in masked fashion, using Metamorph Image Analysis software (Molecular Devices).

The Aca23 mouse eye has been reported to have normal retinal architecture [42]; however, we performed histology on retinal cross sections to check for presence and normal thickness of all retinal layers. We analyzed the histology of the retinas of 4 eyes from 4 animals of each genotype: 4 WT treated retinas, 4 WT untreated retinas, 4 Aca23 treated retinas and 4 Aca23 untreated retinas. After perfusion of the animals and enucleation of the globes, we post-fixed the 16 retinas in 4% paraformaldehyde for 24 h, then in 2% glutaraldehyde/2% paraformaldehyde for 24 h. They were embedded in epoxy resin, cut at 1 µm sections, stained with 1% Toluidine Blue, and examined by light microscopy.

### Statistical evaluation

The parameters that were measured and compared included IOP at baseline and during chronic glaucoma, including IOP average level and IOP exposure over time (positive integral=area under the IOP versus time curve in the treated eye that exceeded the area under the IOP versus time curve in the control eye), axial length and widths, RGC body and axon counts, and parameters of inflation testing. Mean values were compared using parametric statistical tests for data that were normally distributed and median values with non-parametric testing for those whose distributions failed normality testing. Multivariable regression models were used to adjust some comparisons for variables of interest including age, glaucoma status, and strain. Non-parametric ANOVA testing was used to compare stress resultant—strain data among the 3 groups of mice that underwent inflation testing.

## Results

### Intraocular pressure (IOP)

Aca23 homozygotes had mean IOP=13.0±3.9 mmHg (n=122 mice from 4.0 to 20.6 months of age). In a regression model, IOP was significantly higher in older animals (p=0.03, r^2^=0.037, [IOP]=11.86 + 0.12* [Age, months]). Likewise, WT littermate B6 animals had mean IOP=13.0±3.6 mmHg, which rose similarly and significantly with age (p=0.04, r^2^=0.043, n=100 mice from 4.0 to 21.4 months of age, regression equation: [IOP]=11.92 + 0.12* [Age, months]). There was no difference in IOP between Aca23 and WT mice (regression model adjusting for age, p [diagnosis]=0.9, p [age]=0.003, n=222). Baseline IOP measurements for animals in the bead injection experiment showed no significant differences between genotypes or treatment groups (Table 1). In a previous report, we demonstrated that the TonoLab tonometer reads accurately when compared to manometrically determined IOP, both in normal mice and in mice with thin, enlarged corneas [51].

**Table 1:** Baseline IOP Statistics.

**Strain**	**Treatment**	**Mean**	**St Dev**	**Median**
Aca23	Treated	12.11	2.4	13
	Untreated	12.62	2.3	13
WT	Treated	12.37	2.6	13
	Untreated	12.20	3.0	13

No significant differences across genotypes or between fellow eyes in baseline IOP, all p≥0.3, *t*-test.

### Eye Size

Puk et al. [42] demonstrated that 11 week old Aca23 mice have longer anterior chambers and longer eyes than WT B6 mice. We compared Aca23 mice and WT B6 in terms of axial length, and widths in both the nasal—temporal meridian and the superior—inferior meridian. Some eyes were fixed by perfusion and inflated to 15 mmHg by a needle placed in the anterior chamber before measurement. Other eyes were measured unfixed. Some were also exposed to chronic experimental IOP elevation by bead injection. In a multivariable model, axial length was significantly longer in older animals, longer in Aca23 than WT, and longer after exposure to glaucoma, but was not related to method of fixation (Table 2, r^2^=0.58, data from 227 mice). In a similar model, nasal—temporal eye width was significantly larger in older mice, in Aca23 mice, after glaucoma, and after inflation testing and perfusion fixation (p values: <0.0001, 0.0003, <0.0001, 0.02, and <0.0001, respectively, r^2^=0.42). Likewise, superior—inferior width was also significantly longer by age, in Aca23 mice, and after glaucoma (p values <0.0001, 0.04, <0.0001, respectively; r^2^=0.39), but was not related to whether inflation testing had been performed nor to the fixation method employed (both 0.4).

**Table 2:** Regression model for axial length.

** Parameter**	**Coefficient**	**SEM**	**p value**
Age	0.021	0.002	<0.0001
Strain	−0.211	0.014	<0.0001
Glaucoma	0.298	0.028	<0.0001
Inflation testing	0.111	0.044	0.01
Fixation	0.08	0.049	0.1

Coefficient, standard error (SE), and probability (p) values from multivariate regression model.

The Aca23 mice at 4 months of age were 12.2% longer and 7.3% wider than WT, and at an average of 17 months of age they were 16.4% longer and 2.6% wider than the wild type (Table 3; Aca23—WT length and width differences p<0.0001; *t*-test). From 4 months to 17 months of age, Aca23 eyes became 9.7% longer and 3.9% wider (nasal—temporal). WT mice grew 5.7% longer and 8.6% wider during the same age span. Both length and width remained significantly larger in older Aca23 mice than WT mice (p<0.0001 and 0.002, *t*-test).

**Table 3:** Length and width of eyes.

**Strain**	**Age (months)**	**N**	**Control (Mean±SD)**			**N**	**Glaucoma (Mean±SD)**	
x	x	x	**Length (mm)**	**Width SI (mm)**	**Width NT (mm)**		**Length (mm)**	**Width NT (mm)**
Aca23	4	37	3.81±0.10	—	3.51±0.13	37	4.15±0.26	3.74±0.18
Aca23	17	48	4.17±0.12	3.56±0.15	3.65±0.14			
WT	4	127	3.39±0.14	3.23±0.12	3.27±0.14	30	4.01±0.25	3.69±0.29
WT	17	48	3.59±0.11	3.49±0.09	3.55±0.08			

WT=wild type, SI=superior—inferior, NT=nasal—temporal, SD=standard deviation, N=number of eyes, mm=millimeters.

### Inflation test results

Three groups of mice were inflation tested, 29 eyes of 21 Aca23 mice at a mean age of 17 months, 22 eyes of 17 WT B6 mice at a mean age of 18 months, and 20 eyes of 20 WT B6 mice at a mean age of 4 months. The difference in age between the two older groups was not significant (p=0.2, Mann–Whitney test). In the analysis, use of either one eye per mouse or both eyes when both were tested did not alter the conclusions as presented.

**Figure 3:**
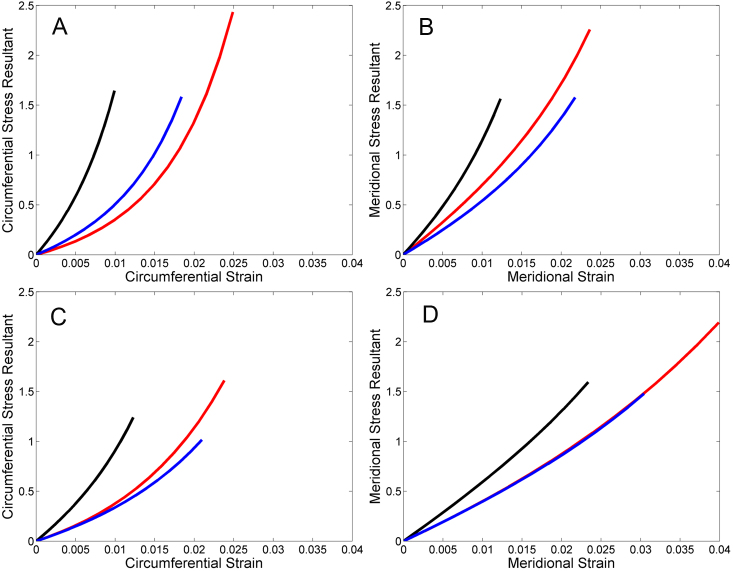
Stress resultant – strain of Aca23, age-matched WT, and younger WT in two different regions and in two different directions. Graph of stress resultants and strains in Aca23 (black), age-matched WT (blue) and younger WT (red) eyes from inflation testing. Data were analyzed from region 2–3 in the circumferential direction (**A**) and the meridional direction (**B**). Likewise, data were analyzed from region 4 in the circumferential direction (**C**) and the meridional direction (**D**). In region 2–3, the Aca23 eyes had significantly lower strain than age-matched and younger WT at several pressure levels in the circumferential and meridional directions. In Region 4, the Aca23 eyes showed significantly lower strain than both WT groups in the circumferential, but not the meridional direction. For statistical analysis, please see Results.

In the more posterior (R2–3) scleral region, strain rose significantly in both circumferential and meridional directions with increasing IOP (both p<0.0001, ANOVA). Aca23 animals had less strain than younger WT animals at all of the pressure steps analyzed from 10 to 30 mmHg in both circumferential and meridional directions (p<0.001, ANOVA; Figure 3A,B). Aca23 eyes also had significantly less circumferential strain than older WT animals from 10 to 26 mmHg (all p<0.05; Figure 3A) and significantly less meridional strain than older WT at 10 and 30 mmHg (p<0.05, ANOVA; Figure 3B). In all 3 groups of animals at this region, circumferential strain exceeded meridional strain significantly at lower IOP, but the difference was reduced at higher IOP (p<0.01, ANOVA).

In the R2–3 region, stress resultants rose significantly with increasing IOP (p<0.0001, ANOVA), but there were no significant differences among the 3 groups in stress resultant values (Aca23 versus younger or older WT, or younger versus older WT).

In the more anterior (R4) scleral region, strain rose significantly circumferentially and meridionally with increasing IOP (Kruskal–Wallis ANOVA p<0.0001; Figure 3C,D). Aca23 eyes had significantly lower strain than age-matched, older WT circumferentially at 4 of the 7 analyzed pressure steps (14 to 26 mmHg; p<0.01, ANOVA Bonferroni corrected; Figure 3C). Aca23 eyes also had lower strain circumferentially compared to younger WT eyes at 6 of 7 analyzed pressure steps (10–30 mmHg; p<0.001; Figure 3C). There were no significant differences between Aca23 and either older or younger WT in meridional strain (Figure 3D). Circumferential and meridional strain did not differ from each other significantly in their response to increasing IOP, nor was this anisotropy comparison significantly different from unity in Aca23, older WT and younger WT animals (ANOVA p=0.9). Strain did not differ between younger and older WT animals in either circumferential or meridional directions (all p>0.05, ANOVA; Figure 3C,D).

In R4, stress resultant values rose significantly with increasing IOP both circumferentially and meridionally (all p<0.0001, ANOVA), but did not differ significantly between Aca23 and older WT, Aca23 and younger WT, nor between younger and older WT.

### Effects of glaucoma

Chronic glaucoma was induced and its effects measured in 37 Aca23 and 30 WT mice beginning at 3.5 to 4.4 months of age, for a period of 6 weeks from injection of beads to sacrifice. Aca23 control eyes were significantly longer than they were wide (p<0.0001, Table 3), while there was no significant length—width difference in WT control eyes (p>0.05, Mann–Whitney test). The Aca23 glaucoma mice increased eye length 9% and width 7% compared to untreated fellow eyes (p<0.05, <0.01), whereas WT mice grew proportionately twice as much, increasing length by 18% and width (nasal—temporal) by 13% (both p<0.001, Mann–Whitney test). After 6 weeks of glaucoma, there was no significant difference in length or in nasal—temporal width between Aca23 and WT mice (both p>0.05).

**Figure 4:**
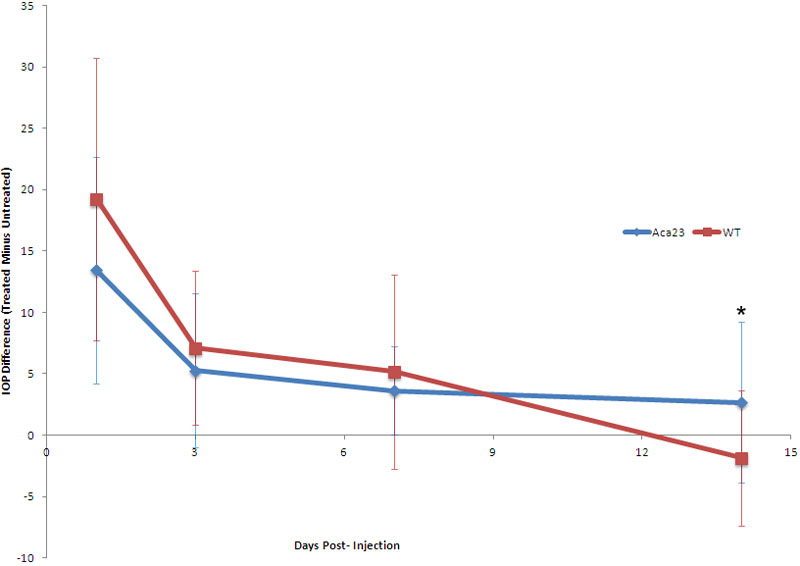
Graph of IOP elevation for 2 weeks after treatment with bead injection. Mean intraocular pressure difference (mm Hg) between injected eye and fellow eye at various times after injection for Aca23 (blue) and wild type (red) mice. Significantly higher mean IOP difference at 14 days in Aca23 group compared to wild type (*p=0.005, Mann–Whitney test); other time periods had no significant mean IOP difference between strains (all p>0.06). Area under curve (positive integral IOP) for two groups not significantly different (see Results Section).

IOP was increased in every bead-injected eye at one or more time points in both Aca23 and WT groups. The mean IOP differences between injected and fellow eyes over the six week experiment are shown in Figure 4, with no significant differences except at 2 weeks where the Aca23 group had higher mean IOP difference. The estimated cumulative IOP exposure was not significantly different between Aca23 and WT, with mean positive integral IOP of 109±127 mmHg—days (Aca23) and 88±79 mmHg– days (WT) and median positive integral IOP=59 and 64 mmHg—days, respectively (p=0.99, Mann–Whitney test).

In 4 month old mice, the number of axons in untreated eyes was 46,905±7,592 in Aca23 and 43,628±11,162 in WT (p=0.43, Mann–Whitney test, n=37 and 29, respectively). In 8 older Aca23 mice, aged 16–18 months, axon number was 51,285±16,106 (p=0.33 comparing Aca23 4 month old to Aca23 16–18 month old, Mann–Whitney test).

**Figure 5:**
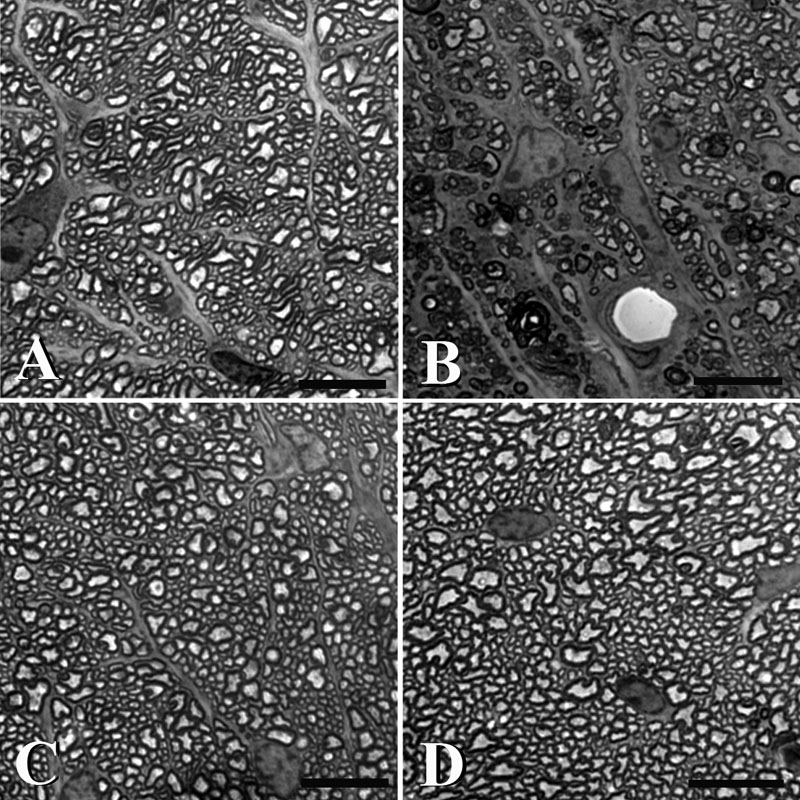
Axon loss in optic nerve cross section. Representative images of optic nerve cross sections stained with Toluidine Blue. **A**: The untreated nerve from a WT animal shows no damage. **B**: Six weeks post intracameral microbead injection, the axons of RGCs show significant loss of axons. **C**: An untreated Aca23 nerve resembles that of an untreated WT animal. However after microbead injection (**D**), the treated Aca23 nerve does not show similar damage to the WT treated nerve (**C**). (scale bar=30 µm).

After 6 weeks of glaucoma, the Aca23 mice lost 0.57±17% of axons, while WT lost 21±31% (median loss: 1% (Aca23) versus 10% (WT), n=37, 29, respectively; Table 4). The greater axon loss in WT mice was significant (p=0.005, Mann–Whitney test; p=0.001, multivariable regression adjusting for positive integral IOP exposure). Representative images of optic nerve cross sections from both genotypes and both treatment conditions are shown in Figure 5.

**Table 4:** Axon counts after experimental glaucoma.

**Strain**	**Treatment**	**Mean**	**SD**	**Mean % loss ±SD**	**Median % loss**
Aca23	Untreated	46905	7592		
	Injected	46638	7987	0.6±17%	1%
WT	Untreated	43628	11162		
	Injected	37025	14650	21±31%	10%

WT=wild type, SD=standard deviation.

**Figure 6:**
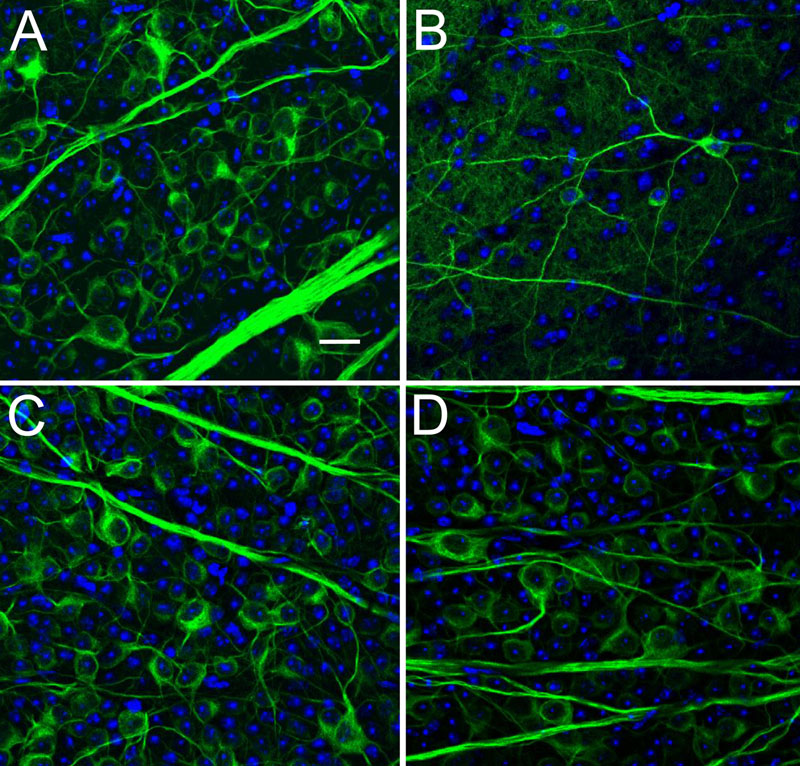
RGC loss in retinal whole mount. Representative images of the RGC layer of retinal wholemounts stained with DAPI (blue) and anti-β-tubulin (green). **A**: An untreated WT control retina with nuclei of all cells including RGC, amacrines and others labeled blue with DAPI. The cytoplasm, dendrites and axons of RGC labeled green for tubulin. **B**: Glaucoma retina from WT animal with substantial loss of RGC. **C**: An untreated Aca23 control retina is similar to untreated WT. **D**: Aca23 retina after glaucoma, showing one of the retinas with modest loss of RGC compared to untreated Aca23 (**C**) or WT (**B**), but less than that of treated WT (**B**). (scale bar=20 µm).

As confirmation of the axon loss measures for RGC damage, we counted RGC bodies identified by anti-β-tubulin antibodies in whole mounts of the retina in 4 retinas of WT and 4 retinas from Aca23 mice, half with experimental glaucoma and half from fellow controls. A total of 2,625 cells were enumerated in these retinas. The mean number of cells identified in the RGC layer of control Aca23 retinas in the selected areas was 256±53 and in control WT retinas it was 227±28 (p=0.3, *t*-test). The median decrease in RGC cell bodies labeled by β-tubulin was 17% in Aca23 eyes and 50% in WT retina (p<0.0001, *t*-test). The corresponding median axon loss for the eyes whose retinas were analyzed was 17% in Aca23 and 41% in WT nerves, showing excellent agreement between the RGC cell body loss and axon loss (Figure 6).

**Figure 7:**
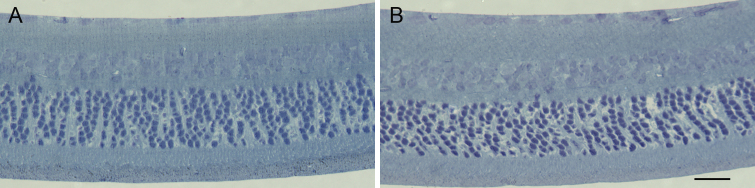
Retinal architecture of Aca23 and WT. Representative cross-section images of the retinas of Aca23 and WT mice before treatment. The Aca23 retina (**A**) has no differences in retinal layer thicknesses from WT (**B**; stained with Toluidine Blue; scale bar=20 µm).

We prepared cross-sections of the retina to confirm normal histology of the retina in both Aca23 and WT eyes. There was normal retinal architecture and normal thickness of retinal layers in both groups in non-glaucoma eyes by phase contrast microscopy (Figure 7). Glaucoma treated eyes had only diminution of RGC layer and nerve fiber layer, but no sign of mid-retinal thinning that would occur if there had been major vascular compromise and inner retinal ischemic atrophy.

## Discussion

Aca23 mice lost significantly fewer RGC with chronic glaucoma than did their WT littermates, with median loss about one-half that of WT. We plan further, detailed studies to identify the reasons for this difference in susceptibility to glaucoma damage. It seems likely that the difference is due to alterations in the structure or behavior of the ocular connective tissues in these mice. At baseline, Aca23 eyes were longer and wider than WT. Our initial hypothesis was that, all things equal, axially longer eyes would show greater glaucoma damage. This hypothesis was based on the fact that human glaucoma is more prevalent and more rapidly progressive in those with longer eyes and eyes with thinner corneas [2]. Theoretically, the scleral stress would be greater at a given IOP in a larger eye than in a smaller eye. Clearly, this simplistic notion does not apply to the situation in the Aca23 eye. In fact, our inflation data showed that Aca23 eyes had less strain at similar stress than control mice in the sclera. Furthermore, while they increased both length and width with chronic IOP elevation, the enlargement was proportionately less than half that of the WT mice. These tendencies to resist stress and to exhibit less fixed deformation may be interpreted to indicate that a stiffer response to IOP was protective against RGC loss.

We have recently completed similar experiments that compare RGC loss in chronic glaucoma between albino CD1 and pigmented B6 mice. CD1 mice have consistently greater damage at the same IOP exposure compared to B6. With inflation testing, the two strains also show significant differences in scleral behavior at baseline and the inflation behavior became stiffer in both strains after chronic glaucoma. Prior to glaucoma, the most significant difference between CD1 and B6 eyes was anisotropy of inflation response between the meridional and circumferential directions. In both strains, the peripapillary sclera thinned, but they differed dramatically in the response of the rest of the sclera. There was uniform scleral thinning in CD1, but actual thickening of sclera in B6 after 6 weeks of glaucoma. We plan to study the detailed response of the sclera in Aca23, CD1, and WT B6 eyes to determine the molecular changes that underlie these differences in susceptibility. Initial proteomic analyses suggest active remodeling induced by chronic glaucoma in pathways linked to transforming growth factor β (unpublished).

Stiffening of the ONH and sclera has been reported in animal glaucoma models and in living and post-mortem human glaucoma eyes. Zeimer et al. [52] reported that the ONH was stiffer in human glaucoma eyes and that the effect was worse with greater RGC damage. Coudrillier et al. [53] compared 24 normal and 11 glaucoma pairs of post-mortem human eyes by inflation testing, determining that glaucoma sclera was thicker and had a stiffer meridional response and slower circumferential creep rates in peripapillary sclera than normal eyes. Testing of living human eyes by indirect methods also suggests that glaucoma eyes have stiffer responses [54,55]. However, it has not yet been determined if human glaucoma eyes have features interpretable as stiffer before damage, or, if their apparently greater stiffness is a result of the disease. Girard et al. [56] studied 9 monkey glaucoma eyes, determining that stiffness increased with moderate glaucoma damage, though the response was variable. Roberts et al. [57] modeled ONH behavior in 3 early glaucoma monkey eyes using connective tissue volume fractions. They hypothesized that scleral stiffening in glaucoma may shield the ONH somewhat by an increased load carried in the sclera. The monkey and mouse experimental glaucoma data suggest that greater stiffness is an effect of glaucoma. Whether greater or lesser compliance of the sclera at baseline alters glaucoma susceptibility is an important area for future investigation. We must keep in mind that, in addition, the response of the sclera to chronically elevated IOP may be as important, or more so, than the baseline state of the tissues. It is feasible to treat the sclera to alter its mechanical stiffness. This approach could represent a new direction for glaucoma treatment, reducing the strain generated by IOP at the ONH.

The decreased susceptibility to glaucoma damage conferred by the Aca23 mutation could be related to features other than those studied as yet. In development, it is hypothesized that collagen 8α2 is a factor in capillary development. Potentially, the mutation could lead to altered nutritional support to RGC and their axons in the retina and ONH that protects these neurons from damage. The expression of collagen 8α2 in astrocytes of the optic nerve has been suggested [36,37]. This raises the possibility that the beneficial effect of the mutation is mediated through glial interactions with RGC. The example of the Marfan syndrome points to the need to think creatively when mutations in structural genes are studied as susceptibility factors in disease. Dietz and colleagues [58] determined that the mutation in fibrillin that leads to protean changes in connective tissues in this disorder does not act by altering the molecular structure of fibrillin itself, but rather by changing a signal sequence for transforming growth factor β. As a result, translational therapy is now being tested to inhibit the overaction of this pathway. Potentially, the effect of collagen 8α2 mutation could be mediated by mechanisms other than simple alterations in molecular structure.

Clearly, the Aca23 mouse exhibits dramatic structural changes in ocular connective tissues. The larger eye and thinner cornea are not an apparent result of exposure to a different IOP level, as these eyes are insignificantly different from control in this parameter. Nor is the phenotype simply a difference in the cornea alone, as the widths of the globe at the equator are significantly different from controls. There are no detectable differences in the number of RGC at baseline, either judged by axon counts or retinal anatomy. This mouse is only one of several strains that we have identified, including those with genetic alterations in elastin and fibromodulin, that lead to larger than normal eyes. It will be interesting to compare how these various models respond to chronic experimental glaucoma.

We have confirmed past studies of age effects in mice [11] regarding their various features, including IOP and eye size. In both mutant and WT mice studied here, there was a small rise in IOP with age. Savinova et al. [59] studied age effects on IOP in a variety of mice using a different measurement technique, detecting either no change or a decline in some species. The mouse eye continues to elongate up to one year of age, and is relatively stable thereafter (submitted). Interestingly, we have found that the thickness of the sclera in the WT B6 strain increases to one year, but then thins substantially in the second year of life. Features that are age-related must be taken into account in animal glaucoma models.

We recognize that several aspects of the present research deserve further study. The inflation studies conducted here did not take into account the local scleral thickness. We have recently developed methods to measure this for incorporation into a more sophisticated, constitutive model of inflation behavior. There may be differences in susceptibility to experimental glaucoma by age. Further studies of Aca23 mice at different ages could be informative.

In summary, a mutation induced in the collagen 8α2 gene produced a larger eye and altered biomechanical behavior on inflation testing. These mice were significantly less susceptible to loss of RGC in an experimental glaucoma model. Study of the role of ocular connective tissues in this and other genetically-altered mouse models could lead to insights into pathogenesis and new therapeutic directions.
